# Geometric morphometrics to distinguish the cryptic species *Anopheles minimus* and *An. harrisoni* in malaria hot spot villages, western Thailand

**DOI:** 10.1111/mve.12493

**Published:** 2020-11-18

**Authors:** K. Chatpiyaphat, S. Sumruayphol, J.‐P. Dujardin, Y. Samung, A. Phayakkaphon, L. Cui, J. Ruangsittichai, S. Sungvornyothin, J. Sattabongkot, P. Sriwichai

**Affiliations:** ^1^ Department of Medical Entomology, Faculty of Tropical Medicine Mahidol University Bangkok Thailand; ^2^ Unité Mixte de Recherche 177‐Interactions Hôte‐Vecteur‐Parasite‐Enironnement dans les Maladies Tropicales Négligées dues aux Trypanosomatidés, Centre International de Recherches Agronomiques pour le Développement (CIRAD) Institut de Recherches pour le Développement (IRD), Campus international de Baillarguet Montpellier France; ^3^ Department of Internal Medicine, Morsani College of Medicine University of South Florida Tampa Florida U.S.A.; ^4^ Vivax Research Unit, Faculty of Tropical Medicine Mahidol University Bangkok Thailand

**Keywords:** *Anopheles harrisoni*, *An. minimus*, geometric morphometric, species complex

## Abstract

*Anopheles minimus* Theobald 1901 and *An. harrisoni* Harbach & Manguin 2007 belong to the same species complex. They are morphologically similar and can exist in sympatry but have blood host preferences. The most accurate method for their identification is based on molecular techniques. Here, we measure the level of interspecific discrimination by geometric morphometry. Sixty‐seven *An. minimus* and 22 *An. harrisoni* specimens were selected based on their morphological integrity and confirmed by identification polymerase chain reaction of internal transcribed spacer 2. These samples were used as reference data allowing for a morphometric identification based on geometric shape. Despite size overlap between the two species, there was a significant shape divergence allowing for differentiation of *An. minimus* and *An. harrisoni* with 90% accuracy. An intraspecific study of *An. minimus* showed a summer period associated to the reducing of wing size, which did not influence the shape‐based differentiation of *An. harrisoni*. Wing venation geometry can be used to distinguish between these cryptic species mainly based on shaped divergence. This study suggests that geometric morphometrics represent a convenient low‐cost method to complement morphological identification, especially concerning damaged specimens, i.e., insects having accidentally lost the anatomical features allowing a reliable morphological identification.

## Graphical abstract

The venation geometry of the wing can be used to distinguish between *An. minimu*s and *An. harrisoni*. Shape‐based discrimination was poorly affected by size variation.
The geometric morphometrics represent a convenient low‐cost method to complement morphological diagnostics, especially concerning damaged specimens.
An intra‐specific study restricted to *An. minimus* showed that there was a reduction in size associated with the hot season, not affecting however the shape‐based differentiation with *An. harrisoni*.

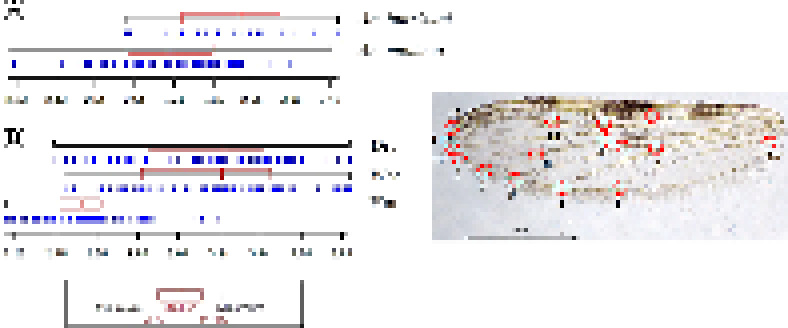

## Introduction

Malaria in Thailand is concentrated along international borders, especially along the Thai–Myanmar border (Sriwichai *et al*., 2016). Major malaria vectors in Thailand, such as *Anopheles dirus*, *An. minimus* and *An. maculatus* all are present in different species complexes; for example, *An. minimus* complex includes other species such as *An. harrisoni* and *An. yaeyamaensis* (Taai *et al*., 2017). *Anopheles minimus* has been incriminated as the main malaria vector in the Greater Mekong Subregion, but the vector status for *An. harrisoni* is unclear. Because of its ability to adapt to and coevolve with vector control strategies, *An. minimus* is distributed throughout Southeast Asia and often occurs in sympatry with *An. harrisoni* (Kengluecha *et al*., 2005; Taai *et al*., 2017). *Anopheles minimus* is an opportunistic feeding on both humans and animals, whereas *An. harrisoni* is more zoophilic (Rwegoshora *et al*., 2002), and in Thailand, it occurs in Kanchanaburi Province (Taai *et al*., 2017). As these malaria vectors differ in their distribution, biting behaviour and capacity for malaria transmission, a low‐cost identification method is an important tool for malaria vector surveillance to evaluate control programs. The routine of vector surveillance system requires to define and monitor the vector species, density and risk of transmission in order to develop effective response to any outbreak/recurrent of vector borne diseases.

Traditionally, mosquito species are identified external diagnostic characters (Sungvornyothin *et al*., 2006; Chan *et al*., 2014). The morphological similarity between *An. minimus* and *An. harrisoni* and their frequent sympatry may often create confusion regarding accurate identification. Misidentification of *An. minimus* has been reported in several past studies (Van Bortel *et al*., 2001; Singh *et al*., 2006; Singh *et al*., 2010) and is most likely due to the use of damaged specimens or to possible phenotypic plasticity of the species (Baba, 1950; Harrison, 1980; Yu & Li, 1984; Sucharit *et al*., 1988; Sucharit & Komalamisra, 1997; Jaichapor *et al*., 2005). Thus, molecular techniques are currently required for their accurate differentiation. Since molecular techniques are more expensive and have to be performed in well‐equipped laboratories (Sumruayphol *et al*., 2016), there is a need for an inexpensive, fast and easy method for accurately identifying species in the *An. minimus* complex. Phenetic techniques such as the geometric morphometrics method have gained popularity in the past decade. This approach is based on shape (e.g., the geometry of wing venation) and, within various species complexes, it has proven effective in supplementing and enhancing morphological identification of mosquitoes (Ruangsittichai *et al*., 2011; Laurito *et al*., 2015; Sumruayphol *et al*., 2016; Chaiphongpachara *et al*., 2019). This method not only can be applied to distinguish species (Lorenz *et al*., 2017) but also can be used to characterize intraspecific morphological variations (Dujardin, 2008). Environmental factors such as relative humidity, food abundance and larval density may produce some shape variation but seem to influence size much more than shape (Morales Vargas *et al*., 2010; Gómez *et al*., 2014). In the recent past, mosquito size was the main morphometric character studied, and consequently, mosquito size has been the topic of many discussions concerning its possible correlation with vector capacity (Dujardin, 2008; Morales Vargas *et al*., 2010; Gómez *et al*., 2014). In our study, we were more concerned with the possible interference of size variation with shape‐based interspecific distinction. This study aimed to explore the power of geometric morphometric analysis of wing venation to accurately distinguish between *An. minimus* and *An. harrisoni*.

## Materials and methods

### Collection of mosquito samples

**Fig. 1:**
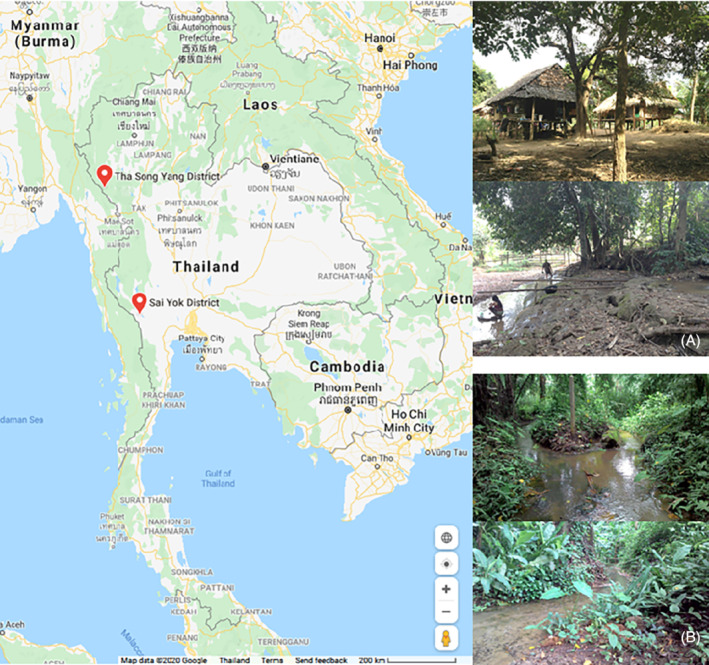
Location of the mosquito collection sites in the red dots; (A) Thasongyang district, Tak province and (B) Saiyok district, Kanchanaburi province, Thailand.

Adult specimens of *An. minimus* were collected from four villages in Komonae (N 17°31′56.9″ E 97°56′55.7″), Suan Oi (N 17°33′28.6″ E 97°55′15.7″), Tala Oka (N 17°19′23.6″ E 98°07′03.4″) and Nong Bua (N 17°20′24.8″ E 98°06′24.2″) in Tha Song Yang District (Tak Province, Thailand) where malaria is endemic (Fig. 1). The Tha Song Yang district is approximately 1920.38 km^2^ (741.46 sq. mi) with a population of 61 161 and density of 31.8/km^2^. The geography is mostly made up of hills and mountains, and the climate is categorized as tropical savanna climate. The villages are located near the Moei River, which separates Thailand from Myanmar, and each village consists of approximately 100 houses. The study sites include rice fields and hilly areas with agricultural plantations.

In 2015, females of *An. minimus* were collected near the houses along forest fringe using Centres for Disease Control and Prevention miniature light traps (BioQuip, USA: model 2836BQ) with a timer rotator (Collection Bottle Rotator model 1512.25, John W. Hock, USA), baited with CO_2_ from dry ice in a bucket. The traps were set up in 30 households in each of the four villages. Mosquitoes were collected for five nights per month from 18:00 to 6:00. Monthly *An. minimus* group collection in 2015 from four villages was detailed in Table S1 and the climatic data of temperature, humidity and rainfall was recorded in Table S2. The season was categorized into summer (March–May), rainy (June–October) and dry cool (November–February) seasons based on rainfall, relative humidity and temperature (Table 1). To allow for an intraspecific study exploring the possible effects of the environment, especially seasons, on size and shape, adult *An. minimus* were randomly selected from summer (*n* = 87), rainy (*n* = 103) and dry cool (*n* = 111) collections (Table 1).

**Table 1:** *An. minimus* sample collection for morphometrics analysis based on season (dry cool, summer, rainy) and village location (Kor Mor Nae (KO), Suan Oi (SO), Tala Oka (TO), and Nong Bua (NB)) in Tak Province, Thailand (2015)

Villages	Dry cool		Summer			Rainy					Dry cool		Total
	Jan	Feb	Mar	Apr	May	Jun	July	Aug	Sep	Oct	Nov	Dec	
KO	‐	4	18	7	9	1	‐	10	9	11	11	33	113
SO	‐	13	16	16	‐	‐	‐	3	6	3	10	1	68
TO	‐	4	3	9	3	‐	‐	11	9	11	11	7	68
NB	‐	4	‐	‐	6	1	‐	7	10	11	11	2	52
Total	‐	25	37	32	18	2	‐	31	34	36	43	43	301

The mosquitoes were collected every morning, placed in a plastic Petri dish with filter paper, and labelled (e.g., trap number, type and location). The samples were stored in a Petri dish under −20 °C for morphological identification.

*Anopheles harrisoni* was harvested by simply collecting larvae from a specific breeding area with 72% humidity and a temperature of 29.9 °C in Ban Pu Toei village (N 14°20′09.5″ E 98°59′20.9″ 296 m elevation), Sai Yok district (Kanchanaburi Province, Thailand). The study site, which is located in a valley between mountains with decaying plantations, consists of dense forests and slow‐running streams (Fig. 1). Selection of the site was based on a recent report indicating that *An. harrisoni* was present in Kanchanaburi but not in Tak (Taai *et al*., 2017). Larvae were collected from these streams in July 2017 with a dip netting method and were transported in a plastic bottle (600 mL) to the laboratory. They were reared in plastic trays with filtered water mixed with the water from the collection site to prevent drastic changes in water conditions. The larvae were kept in an insectary (25 °C, 80% relative humidity, 12 h light) and fed with ground fish food twice a day in the morning (7:30) and afternoon (15:30). Once the larvae developed to the pupal stage, they were transferred to plastic cups for adult emergence. The adult mosquitoes were fed with cotton buds soaked with 10% sugar and vitamin C. They were used for morphological identification 5 days after emergence.

### Selection of reference specimens

**Fig. 2:**
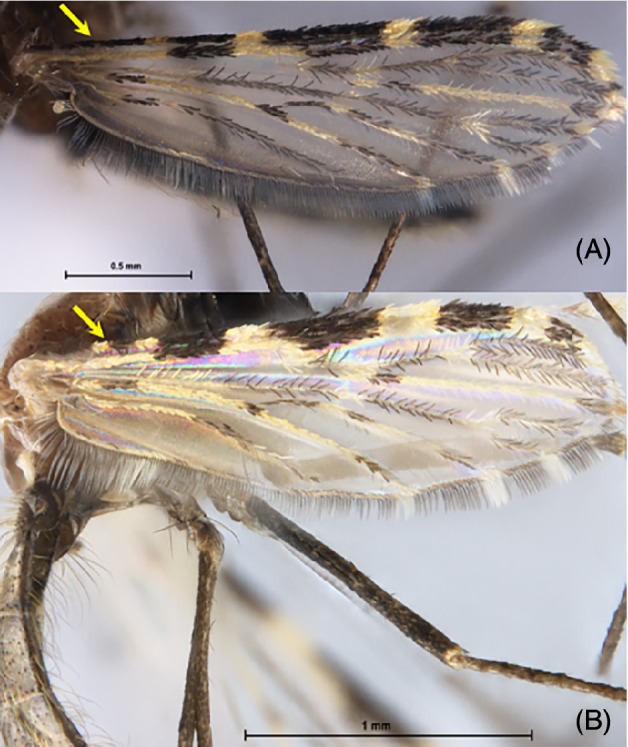
The morphological character used to distinguish between *An. minimus* (A) and *An. harrisoni* (B) is the presence of a humeral pale spot (HP) that is only present in *An. harrisoni* (arrow). The specimens having accidentally lost this small area of the wing are said by us to be “damaged”.

The mosquitoes selected for the interspecific study (Table 2) were those possessing an unambiguous morphological identification using the keys described earlier (Rattanarithikul *et al*., 2006). Currently, the main distinguishing characteristic is the presence (*An. harrisoni*) or the absence (*An. minimus*) of a humeral pale spot located at the costa of the wing (Fig. 2) (Sungvornyothin *et al*., 2006). The specimens having accidentally lost this diagnostic feature are said by us to be “damaged”. Thus, 67 non‐damaged specimens of *An. minimus* were retained for the study, which covered the three seasons and the four villages (Table 2). Since they were reared and emerged in laboratory conditions, the 22 *An. harrisoni* specimens did not suffer any damage due to capture and transportation.

**Table 2:** Female selected samples of *An. minimus* mosquitoes for multiplex PCR analyses in Tak Province, Thailand (2015)

Villages	Dry cool		Summer			Rainy					Dry cool		Total
	Jan	Feb	Mar	Apr	May	Jun	July	Aug	Sep	Oct	Nov	Dec	
KO	‐	1	2	4	1	1	‐	2	2	2	2	2	19
SO	‐	2	1	3	‐	‐	‐	1	2	1	2	1	13
TO	‐	4	2	2	0	‐	‐	1	2	2	2	3	18
NB	‐	4	‐	‐	2	1	‐	2	2	2	2	2	17
Total	‐	11	5	9	3	2	‐	6	8	7	8	8	67

*Note*: The mosquitoes were collected in four villages of the Tak province: Kor Mor Nae (KO), Suan Oi (SO), Tala Oka (TO) and Nong Bua (NB).

To confirm the morphological identification of the selected specimens, genomic DNA was extracted from the hind leg of the selected subset 67 adult specimens using a QIAamp® DNA Mini Kit for DNeasy Blood & Tissue Kit (Hilden, Germany). Multiplex PCR was performed using three sets of primers (ITS2A, MIA, and MIC) as described previously (Garros *et al*., 2004). The ITS2A serves as the universal forward primer (TGTGAACTGCAGGACACAT), whereas species were differentiated using the species‐specific reverse primers for *An. minimus* (CCCGTGCGACTTGACGA) and *An. harrisoni* (GTTCATTCAGCAACATCAGT). The PCR contained a final volume of 25 μL, which included 2.5 μL of 1 × PCR buffer (Invitrogen), 0.75 μL of 50 mM MgCl_2_, 0.5 μM of 10 μM deoxyribose nucleotide triphosphate, 0.3 μL of 10 μM for each primer, 0.2 units of *Taq* polymerase (Invitrogen), and 1 μL of DNA template. The amplification profile was one cycle at 94 °C for 2 min, followed by 40 cycles of denaturation at 94 °C for 30 s, annealing at 50 °C for 30 s, extension at 72 °C for 40 s, and an additional cycle of final extension at 72 °C for 5 min. For each reaction, 4 μL of the PCR product was subjected to 2% agarose gel electrophoresis and stained with SYBR® Safe Stain. The expected PCR product size is 310 bp in *An. minimus* and 180 bp in *An. harrisoni*.

### Geometric morphometric data

**Fig. 3:**
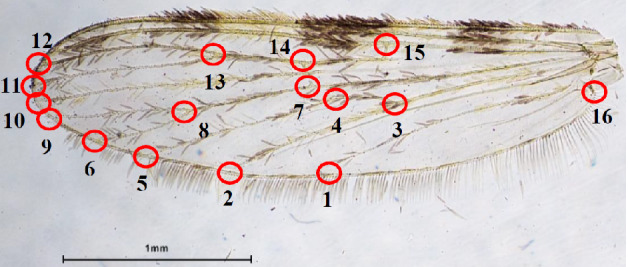
The 16 anatomical landmarks selected for the geometric morphometric analyses. The unit of the wing scale is in millimetres.

The left wings of the 89 (67 + 22) selected specimens were removed and mounted onto a slide using Hoyer medium (Schauff, 2001) for imaging and landmark digitization. The wing images were taken using the NES‐Elements D 3.2 software and a manual microscope (Nikon Eclipse 6600, Japan) connected with a digital camera (Nikon Digital Sight DS‐Ri1, Japan). Sixteen type I landmarks (Bookstein, 1991) were used (Fig. 3) according to previous descriptions (Sumruayphol *et al*., 2016). The scale unit of each wing image was 1 mm.

### Statistical analysis

The CLIC package version 99 was used for statistical analysis (https://xyom‐clic.eu/the‐clic‐package/). Wing metric data were collected as *x* and *y* coordinates and transformed using generalized Procrustes analysis (i.e., they were corrected for size to focus on shape and for position and orientation to remove non‐biological changes). The resulting shape variables were called partial warps (PW); the final shape variables were represented by relative warps (RW), which are the principal components of the PW (Rohlf & Slice, 1990).

The global size of the wing was computed as centroid size (Bookstein, 1991), and the units of centroid size, originally pixels, were converted to millimetres (Ruangsittichai *et al*., 2011). Size and shape variables were analysed separately, and their possible relationship, or allometry, was measured by linear regression techniques. The statistical significance of size difference between groups was estimated using a non‐parametric test with 1000 between‐group permutations, and pairwise post hoc analysis with the *P* value of 0.05.

The discriminant analysis (DA) used to separate species was performed using the set of the first RW adapted to the sample size as input. The DA was illustrated by a histogram showing species classification along with the unique discriminant factor. The Mahalanobis distance was used as a measure of shape divergence between groups, and statistical significance was based on permutation tests (1000 runs) with the *P* value of 0.05. Cross‐check (also called validated) classification scores based on shape variation (thus, excluding size variation) were calculated using the jackknife procedure (Manly, 2004).

To evaluate possible allometric effects on shape variation (i.e., to determine to what extent changes in size could contribute to the shape divergence observed between species), the linear correlation coefficient was calculated between size and the unique discriminant factor derived from shape variation (Dujardin, 2008).

To ensure that digitization was consistent throughout the study, the 67 *An. minimus* and 22 *An. harrisoni* wings were digitized twice. A repeatability test was then performed as an indirect estimate of the error in measuring the wing size and shape (Arnqvist & Märtensson, 1998). A global estimator of repeatability for shape was obtained using the Procrustes ANOVA (Klingenberg & McIntyre, 1998).

## Results

### Selection of reference material

Morphological identification of females of *An. minimus* was based on the lack of the humeral pale spot (Fig. 2), and all originated from Tak Province, whereas *An. harrisoni* specimens from Kanchanaburi, unequivocally possessed the humeral pale spot present. Multiplex PCR based on ITS2 amplification confirmed that all 67 samples collected in Tak Province were *An. minimus* (product size 310 bp), whereas all 22 samples collected in Kanchanaburi were *An. harrisoni* (product size 180 bp) (Garros *et al*., 2004).

### Morphometric characterization of species

**Fig. 4:**
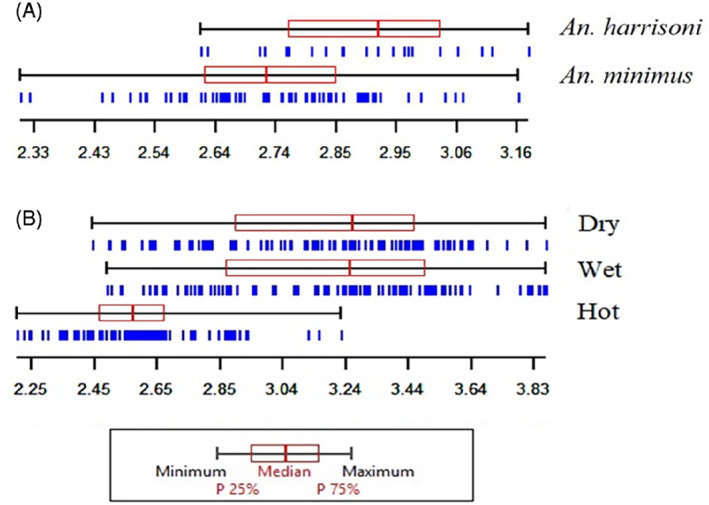
Box plot of the wing centroid sizes (CS) using landmark‐based geometric morphometric analysis. Variation of wing centroid sizes (converted from pixels to millimetres): (A) between *An. harrisoni* and *An. minimus* and (B) among *An. minimus* collected during the summer, rainy and dry cool seasons from Tak Province. Each box is the group median that separates the 25th and 75th quartiles. Vertical blue bars represent the individual specimens.

**Fig. 5:**
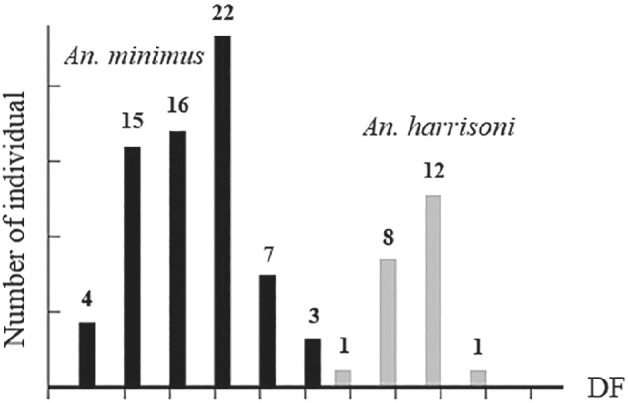
Discriminant analysis between *An. minimus* (black bar) and *An. harrisoni* (grey bar). The distance between each bar represents the difference in wing shape, and the numbers represent the amount of individual samples. X axis represents discrimination factors (DF). Y axis displays individual sample numbers.

**Fig. 6:**
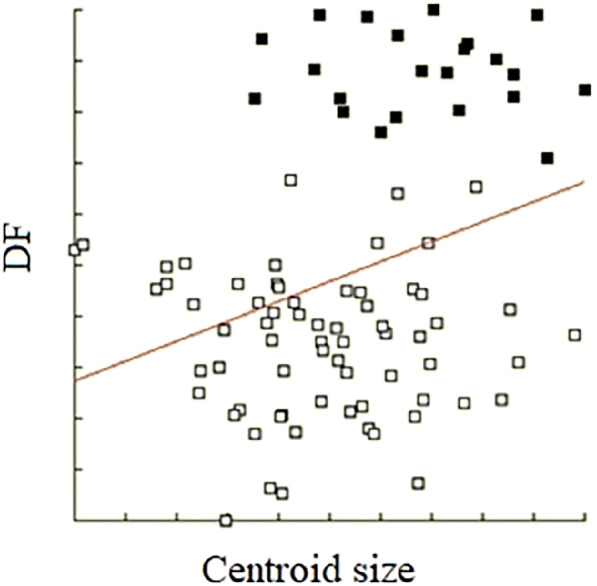
The size effect still included in the shape‐based discriminant factor. The determination coefficient was 9% (*P* = 0.000; *t*‐test = 3.090) between diverging shapes (DF, or discriminant factor) and sizes (centroid size). The open squares represent individual *An. minimus* samples and the filled squares are *An. harrisoni*.

The repeatability score of *An. minimus* was 99.56% for centroid size and 93.17% for shape. The repeatability score of *An. harrisoni* was 99.57% for centroid size and 88.49% for shape. The mean centroid size of *An. minimus* (2.73 mm) was significantly smaller than that for *An. harrisoni* (2.89 mm); however, the range of size variation in the two species significantly overlapped (Fig. 3A). The DA showed an almost complete and significant separation of shape between *An. minimus* and *An. harrisoni* (Fig. 4). The discriminant analysis showed an almost complete and significant separation of shape between *An. minimus* and *An. harrisoni* (Fig. 5). The total cross‐check classification score of shape‐based species diagnostic was 90% (80/89), 88% (59/67) for *An. minimus* and 95% (21/22) for *An. harrisoni*. The influence of size variation on this shape‐based diagnostic was 9% (Fig. 6).

### Geographic and climatic subpopulations of *Anopheles minimus*

The mean centroid size of *An. minimus* for the summer was 2.62 mm, significantly smaller than the 3.19 mm value observed in the rainy and cool dry seasons (Fig. 3B). The wing shape showed significant differences between seasons with p‐values ranging from 0.001 to 0.023. Despite this significant shape variation, it was not possible to accurately classify the wings according to the seasons and the classification score ranged between 37% and 54%.

## Discussion

*Anopheles minimus* and *An. harrisoni* are two cryptic species often found in the same locations in western Thailand (Sungvornyothin *et al*., 2006). The unambiguous morphological distinction between these species requires non‐damaged specimens; therefore, molecular techniques may be necessary in case of doubt.

PCR‐based techniques commonly represent the most reliable diagnostic tools. Since more than one genetic marker may be needed to differentiate multiple species in a complex, the multiplex PCR technique may be required (Garros *et al*., 2004). For instance, the D3 domain of the 28S rDNA failed to distinguish *An. harrisoni* from *An. fluviatilis* (Singh *et al*., 2006), although this marker is the primary marker to discriminate between *An. minimus* and *An. harrisoni* (Sharpe *et al*., 1999; Harbach, 2019). Currently, multiple molecular markers from both ribosomal genes and mitochondrial DNA are available to distinguish *An. minimus* from *An. harrisoni* (Harbach *et al*., 2007).

Our molecular analyses confirmed the validity of the diagnostic character described by Rattanarithikul *et al*. (2006) (i.e., a humeral pale spot located at the costa of the wing). However, this small tuft of white scales is labile and can easily be lost in damaged specimens, making it difficult or impossible to recognize the species. Here, we selected two samples of unquestionably confirmed specimens of *An. minimus* and *An. harrisoni* and used them as reference data to evaluate the diagnostic power of the geometric technique approach.

The morphometric method applies after a wing preparation procedure involving slide mounting and imaging preparation. Skills in these aspects are common among entomologists and do not represent procedural issues (Sumruayphol *et al*., 2016; Chaiphongpachara *et al*., 2019).

The two species were distinguished using shape variables describing the venation geometry of the wings. The confirmed identification produced satisfactory results and showed that 90% of females were correctly identified using the wing shape. In contrast, the wing size failed to identify the species. Results of the allometric analyses demonstrated that the variables describing shape were not completely free of size effects. Such a size effect, up to 9% in this study, could be involved in the evolutionary divergence between the two species (Sharpe *et al*., 1999; Rwegoshora *et al*., 2002) and could also be due to some environmental variation (Dujardin, 2008; Morales Vargas *et al*., 2010).

Our *An. minimus* and *An. harrisoni* samples were not strictly sympatric; therefore, size variation between samples was expected (Phanitchat *et al*., 2019). Because of passive allometric effects, size variation may have an impact on shape variation; this begs the question, to which extent do different geographic origins affect the interspecies divergence of shape? An indirect answer was provided by our study regarding seasonal influence on *An. minimus* metric properties, i.e., size and shape. The temperature (in summer) indeed affected size and shape, but it could not produce shape divergence important enough to allow for the reclassification of individuals according to the seasons. Thus, our intraspecific study of *An. minimus* illustrated what can be expected between different geographic areas: a clear effect on size and a poor effect on shape (Dujardin, 2008; Morales Vargas *et al*., 2010).

Another potential bias affecting our study could be the different procedures of collection for the two species: *An. minimus* was collected in the field at the adult stages, whereas *An. harrisoni* was collected at larval stages and examined after emergence in our laboratory. These distinct procedures did not mean that our interspecific comparisons also included another one between field and laboratory environments. Since morphogenesis is developing during immature stages, and not during emergence, the adult laboratory F1 specimens were not expected to develop shape changes.

In practice, the most important bias when comparing shapes could very well be the digitization step. When collecting the relative positions of the anatomical landmarks of the wings, one must be careful not to introduce artefactual variability due to excessive digitizing error. This potential source of error is especially important between different users (Arnqvist & Märtensson, 1998); therefore, comparative analyses are recommended to be performed by a single user (Dujardin *et al*., 2010). Even under the control of a unique user, digitizing error might be too high, especially for mosquito wings covered by scales, which could hide veins intersections (Garros & Dujardin, 2013). Our samples were digitized twice, showing that the measurement error could range from 6% to 11%. Despite these relatively high values, the total diagnostic power of the method was high (90%).

## Conclusion

The two cryptic species, *An. minimus* and *An. harrisoni*, had overlapping sizes but possessed divergent shapes regarding their wing venation. Our study demonstrated that this feature could be used to distinguish between them. This inexpensive and simple method represents an effective technique to confirm throughout the year the identity of damaged specimens, i.e., insects having accidentally lost the anatomical features allowing a reliable morphological identification.

GPA: generalized Procrustes analysis
DA: discriminant analysis
RW: the relative warps
PW: the partial warps
CS: centroid size
HP: humeral pale spot
DF: discrimination factors
ITS2: internal transcribed spacer 2
PCR: polymerase chain reaction
MgCl_2_
: magnesium chloride
dNTP: deoxyribose nucleotide triphosphate
μL: microliter
μM: micromolar
°C: degree Celsius
mL: millilitre
mm: millimetre
h: hour
bp: base pair

## Ethics approval and consent to participate

The study was approved by the Faculty of Tropical Medicine‐Institute Animal Care and Use Committee (FTM‐ACUC 026/2018) Mahidol University, Thailand.

## Author contributions

PS, JPD, LC, and JS contributed to overall study design. KC, SS, YS, AP, and JR identified the study location, mosquito collection, and confirmation of vector species. KC, SS, PS, and JPD analysed the data. KC, PS, SS, JPD, JS, and LC drafted and revised the manuscript. All authors read and approved the final manuscript.

## Supporting information

**Table S1**: Monthly *An. minimus* group collection in 2015 from four villages in Tha song Yang district, Tak province.
**Table S2**: Climatic data of temperature, humidity, and rain fall in 2015 in Tha Song Yang district, Tak province.

## Data Availability

The datasets used and/or analyzed during the current study are available from the corresponding author upon reasonable request.
