# Supplementary material for: Geometric morphometrics to distinguish the cryptic species Anopheles minimus and An. harrisoni in malaria hot spot villages, western Thailand
Source: Med Vet Entomol. 2020 Nov 18;35(3):293–301. doi: 10.1111/mve.12493 (PMC8451769; doi:10.1111/mve.12493)
Supplement: Supplementary file 1 — Table S1: Monthly An. minimus group collection in 2015 from four villages in Tha song Yang district, Tak province. Table S2: Climatic data of temperature, humidity, and rain fall in 2015 in Tha Song Yang district, Tak province. [file MVE-35-293-s001.docx]

**Supplementary data**

**Table S1** Monthly *An. minimus* group collection in 2015 from 4 villages in Tha Song Yang district, Tak province.

| **Year 2015** | **Jan** | **Feb** | **Mar** | **Apr** | **May** | **Jun** | **Jul** | **Aug** | **Sep** | **Oct** | **Nov** | **Dec** | **Grand Total** |
| --- | --- | --- | --- | --- | --- | --- | --- | --- | --- | --- | --- | --- | --- |
| Left | 0 | 24 | 38 | 18 | 10 | 3 | 1 | 53 | 80 | 57 | 42 | 26 | 352 |
| Left & Right | 0 | 24 | 38 | 32 | 18 | 2 | - | 89 | 656 | 181 | 125 | 61 | 1226 |
| Right | 0 | 25 | 44 | 13 | 10 | 2 | - | 36 | 102 | 68 | 56 | 31 | 387 |
| Grand Total | 0 | 73 | 120 | 63 | 38 | 7 | 1 | 178 | 838 | 306 | 223 | 118 | 1965 |

**Table S2** Climatic data of temperature, humidity, and rain fall in 2015 in Tha Song Yang district, Tak province.

| **Month** | **Jan** | **Feb** | **Mar** | **Apr** | **May** | **Jun** | **Jul** | **Aug** | **Sep** | **Oct** | **Nov** | **Dec** |
| --- | --- | --- | --- | --- | --- | --- | --- | --- | --- | --- | --- | --- |
| Temperature (Celsius) | 22.2 | 24.5 | 27.9 | 29.2 | 29.5 | 27.5 | 26.2 | 26.2 | 26.8 | 26.7 | 27.0 | 25.0 |
| Mean of rain fall (mm) | 66.6 | - | 24.9 | 36.8 | 106.5 | 224.4 | 465.3 | 296.2 | 209.1 | 120.6 | 1.5 | 11.3 |
| Relative humidity (%) | 71.0 | 63.0 | 65.0 | 65.0 | 76.0 | 85.0 | 89.0 | 89.0 | 87.0 | 82.0 | 75.0 | 73.0 |
